# Supplementary material for: Molecular mechanisms of genotype-dependent lifespan variation mediated by caloric restriction: insight from wild yeast isolates
Source: Front Aging. 2024 Jul 11;5:1408160. doi: 10.3389/fragi.2024.1408160 (PMC11269085; doi:10.3389/fragi.2024.1408160)
Supplement: Supplementary file 3 [file DataSheet1.pdf]

Supplementary Figures

McLean et al., 2024:

Molecular Mechanisms of Genotype-Dependent Lifespan Variation Mediated by Caloric Restriction: Insight from Wild Yeast Isolates

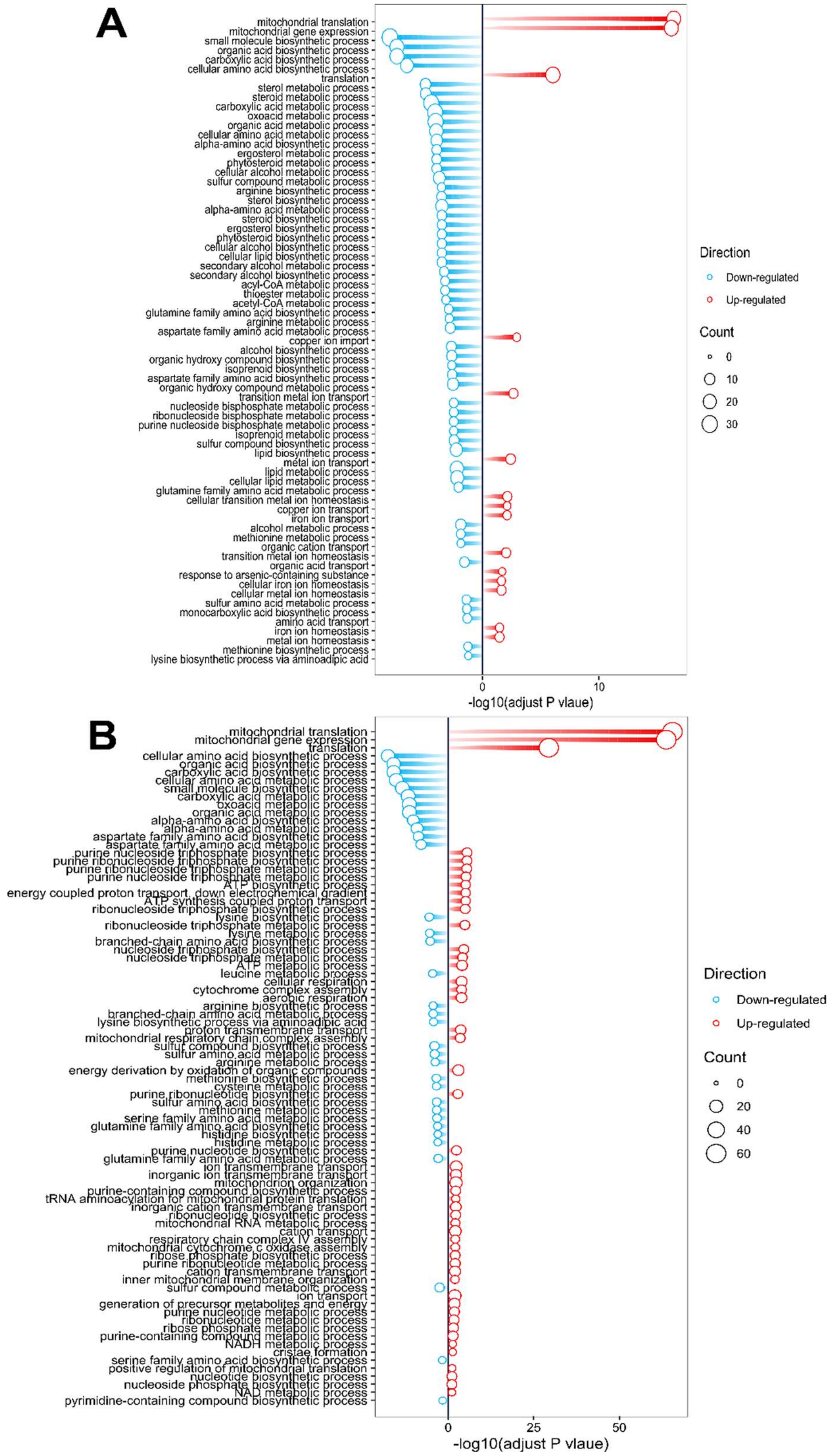

Figure S1: GO term enrichment analyses: The GO term (Molecular Function) analyses show significantly enriched terms for (**A**) positively responding versus non-responding and (**B**) positively responding versus negatively responding strains.

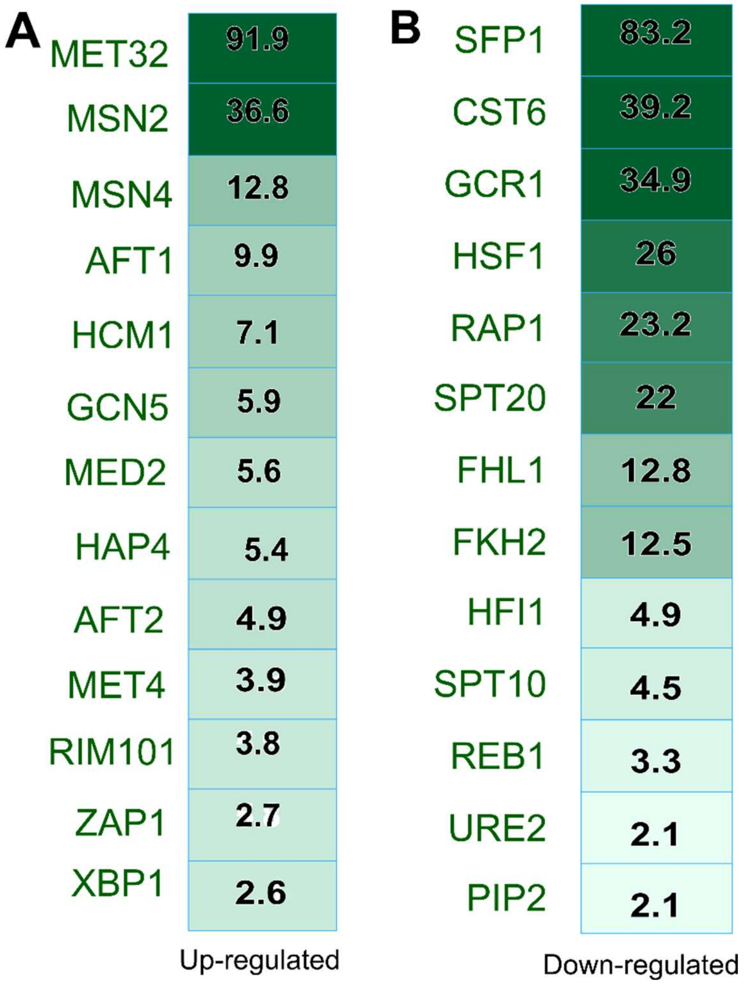

Figure S2. Transcription factor enrichment analyses for non-responding strains analyzed under CR condition. Significantly enriched TFs for (**A**) up- and (**B**) down-regulated DEGs obtained under CR conditions for non-responding strains. The color index and the number show the significance of the enrichment score (-log 10 , adjusted P-value).

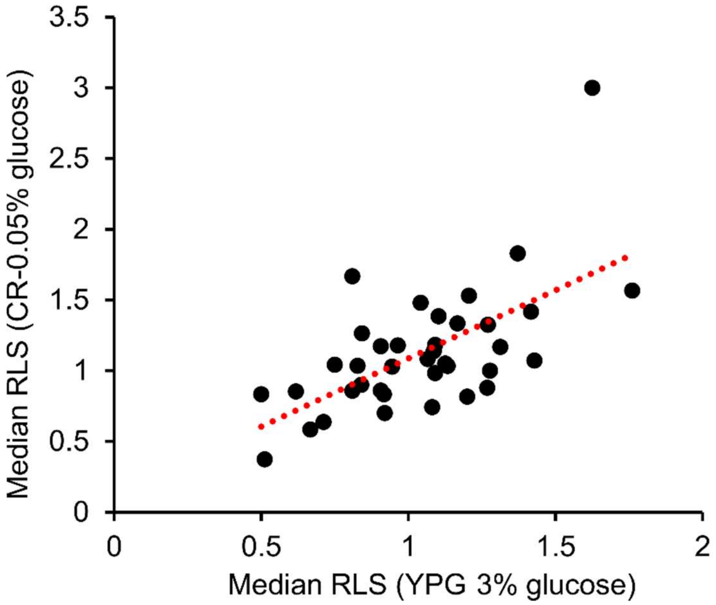

Figure S3. A positive correlation of median RLS phenotype between YPG and CR medium was observed. A significant correlation was observed between median RLS in YPG and median RLS in CR, wherein glycerol-induced CR-induced respiration approximately equally extends the RLS of those strains we tested (R 2 = 0.62, Padj value = 2.29x10-4).

Supplementary Files:

Supplementary file 1: The file includes the strain list used in this study and their replicative lifespan analyzed under YPD, YPG, and CR conditions, along with the statistical significance of lifespan differences.

Supplementary File 2: Differentially expressed genes and metabolite abundances resulted from comparison of responding (POS) versus non-responding (NON) and negatively (NEG) responding comparisons. These -omics data were obtained under YPD conditions. The file also contains results from the regression analysis for the genes and metabolites whose transcript level/abundances correlate with CR-lifespan. The file also includes results from the GO term enrichment analysis associated with Figures 9 and 10.

Supplementary File 3: The file includes raw and normalized counts for RNA-seq data for selected responding and non-responding strains. Differentially expressed genes for each group resulting from comparison to the control (YPD) are also included.
